# In Vitro Exploration of Probiotic Bacteria Interactions with *Candida* Using Culture Techniques to Model Dysbiotic Conditions in Colonized Tissues

**DOI:** 10.3390/pathogens10030289

**Published:** 2021-03-03

**Authors:** Emma Wittman, Neela Yar, Francesco De Seta, Bryan Larsen

**Affiliations:** 1 College of Osteopathic Medicine, Marian University, Indianapolis, IN 46222, USA; ewittman930@marian.edu (E.W.); nyar856@marian.edu (N.Y.); 2 Department of Medical Sciences, University of Trieste, Institute for Maternal and Child Health-IRCCS, Burlo Garofolo, 34137 Trieste, Italy; Francesco.deseta@burlo.trieste.it

**Keywords:** vaginal microbiome, *Candida albicans*, *Lactobacillus*, co-culture, flow cytometry

## Abstract

*Candida albicans* overgrowth at various mucosal sites is an ongoing and complex clinical concern involving interactions with indigenous microbiota and therapeutic or preventive measures superimposed on the pathogen-microbiome interaction. In this paper we describe the use of quantitative flow cytometry (specific to the cytometer’s sample introduction mechanism) to explore the in vitro interaction between *Candida albicans*, probiotic lactobacilli and a topical vaginal therapeutic. Our central hypothesis was cytometric measurements of co-cultures of yeast and bacteria could provide a useful method for exploring the dynamics of different microbial species in culture, with and without inhibitors. Two commercial products were used as exemplars for this research, a vaginal antimicrobial gel and two species of probiotic lactobacillus intended or oral administration with crystalline bovine lactoferrin to augment the vaginal gel. The cytometer forward channel height parameter distinguished yeast from bacteria in co-culture experiments in the presence of a vaginal therapeutic gel or components of its formulation including EDTA, glycogen, polydextrose as well as the host defense factor, lactoferrin. Flow cytometry showed lactobacilli influenced yeast counts in co-culture, with the technique lending itself to wide-ranging test conditions including organisms, media composition and screening of various antimicrobials. Key findings: The proprietary vaginal gel augmented the effect of lactobacilli, as did EDTA and lactoferrin. Prebiotic compounds also enhanced *Candida* inhibition by lactobacilli. Propidium iodide (Fluorescence channel 3) discriminated between necrotic and non-necrotic yeast and bacteria in co-cultures under various culture conditions. This research demonstrates the value of flow cytometry to evaluate the population dynamics of yeast and bacteria in co-culture using a proprietary product and its components. We discuss both the limitations of the current study and describe how methods employed here would be transferrable to the investigation of organisms present in defined cultures or at body sites colonized by fungal species and the effects of therapeutics or probiotics on *Candida*.

## 1. Introduction

*Candida albicans* colonizing the vaginal tract may overgrow under certain circumstances to produce symptoms frequently seen in gynecologic practice. Typically, this is addressed by topical or enteral antifungal drug therapy. The vaginal microbiome has been demonstrated to influence *Candida* colonization, with *Lactobacillus crispatus* being associated with a non-permissive state compared to *Lactobacillus iners* (less permissive) and the diverse microbiome with low *Lactobacillus* colonization being most permissive for *Candida* colonization [1] leading to interest in using probiotic lactobacilli as therapeutic or preventive measures [2] particularly if there is a desire to limit use of antimycotic pharmaceuticals which may select of drug-resistance.

Major research enterprises in industry or academic medical centers are using high throughput methods to perform genomic, proteomic and metabolomic studies addressing some of the questions related to benign colonization and opportunism in animal and human subjects and, applying these contemporary multi-omic methods to elucidate the composition and dynamics of the microbiome under various circumstances. Such methods demand a significant infrastructure and may not be accessible to smaller laboratories. However, we assert there remains a place for functional studies involving microbial interactions in controlled environments using less elaborate methods, especially as a prelude to selecting probiotic agents and antimicrobial compounds to test therapeutic potential. We have been using flow cytometry to evaluate growth and growth inhibition of *Candida* and consider it potentially useful in evaluating mixed cultures of bacteria and yeast which supports simultaneous monitoring of the growth of yeast and probiotic bacteria under various experimental conditions.

Not surprisingly, probiotic bacteria have been considered as a potential alternative or adjunct to pharmaceutical drugs for vaginal symptoms and considerable literature has developed around this concept as recently reviewed [3]. In a 2017 systematic review, authors lamented that the quality of 10 randomized clinical trials reviewed, proved less robust than for studies of pharmaceutical drugs [4].

In papers published by Russo et al., two probiotic strains of *Lactobacillus* (*L. acidophilus* and *L. rhamnosus*) combined with bovine lactoferrin were used as an oral adjunct to standard drug treatment of either vaginal candidiasis [5] or bacterial vaginosis [6]. In addition to improving clinical efficacy, the orally administered probiotic bacteria were culturable from the vaginal specimens of subjects. This information was the basis for experiments to evaluate utility of flow cytometric analysis in the context of a proprietary vaginal gel and probiotic organisms intended to augment its activity against *Candida.*

*L. acidophilus, L. rhamnosus* and bovine lactoferrin, as well as the vaginal gel product and its active components were evaluated for interaction with a panel of *Candida albicans* organisms which included community isolates and drug-resistant strains. This work was particularly concerned with possible enhancement of the vaginal gel activity against *Candida* by probiotic lactobacilli and vice versa and exploited the cytometric techniques for this work. The vaginal gel used in this study is a product of Giellepi SpA, Italy, marketed as Respcta^®^ BalanceGel (abbreviated hereafter as “R”) and includes components (see methods) that may influence interactions between *Candida* and probiotic organisms.

## 2. Results

### 2.1. Monoculture in Spent Medium

**Figure 1:**
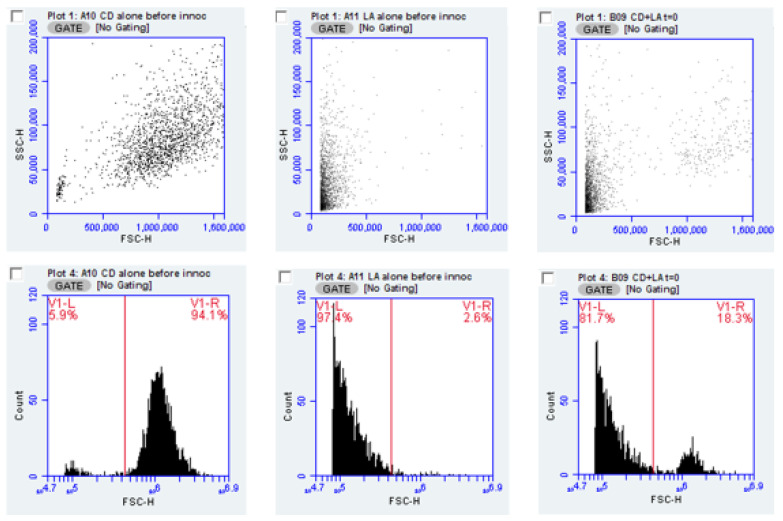
Data for flow cytometer-based counting and differentiation of yeast and bacteria are presented as screens from flow cytometry analysis. Upper panels depict scatter plots for (left to right) yeast, bacteria and a mixture of yeast and bacteria. The lower panes show the identical FSC data as histogram plots and a marker to provide readout of the distribution between yeast and bacteria. By convention, the cytometer software defaults to an arithmetic scale for FSC vs. SSC counts and a logarithmic scale for histogram plots. In this analysis the scatter plot with yeast only contains some signal in the area where bacteria occur, due to electronic background noise and these counts are miniscule compared to bacterial counts and may be subtracted from the counts attributed to bacteria.

To demonstrate the ability to measure bacterial and yeast content of cultures, pure cultures of *C. albicans* and *L. acidophilus* or *L. rhamnosus* from overnight culture were diluted ten-fold in water and evaluated by flow cytometry. Figure 1 illustrates results obtained from these test organisms, individually and in combination. As the forward scatter parameter (FSC) indicates the relative size of the particles (bacteria or yeast) in the sample, this measure allows, with certain cautions and caveats, discrimination between species used in this experiment; the FSC parameter by itself, would not be suitable for distinguishing two bacterial species in mixed culture. However, we suggest strategies for extending this method using specific fluorescent stains (see Discussion). In Figure 1, the data from the FSC vs. Side scatter (SSC) parameters form the basic plot used to trigger the counting of individual particles (bacteria or yeast). The cytometer events for the same data can be presented as a histogram, which plots the events logarithmically and may allow a less subjective placement of the marker. Plotting the by histogram and setting a marker, shows the percent of the population represented by yeast or bacteria as seen in the figure. The FSC value where the marker is placed is at 230,000.

FSC signal amplitude may be used to underscore how distinction is made between bacteria and yeast. Mean forward scatter height for six cultures of each of the two *Lactobacillus species* and four cultures of each of three *Candida* strains yielded FSC average height values of 0.136 × 10^6^ for *L. acidophilus* and 0.125 × 10^6^ for *L. rhamnosus*. For *C. albicans* strains CA1, CAA and CAD, mean FSC-H values were 1.37 × 10^6^, 1.2 × 10^6^ and 1.25 × 10^6^, respectively. Thus, FSC amplitude values were an order of magnitude different between bacteria and yeast (for reference, the dynamic range of FSC values for the cytometer is about 6.5 decades).

**Figure 2:**
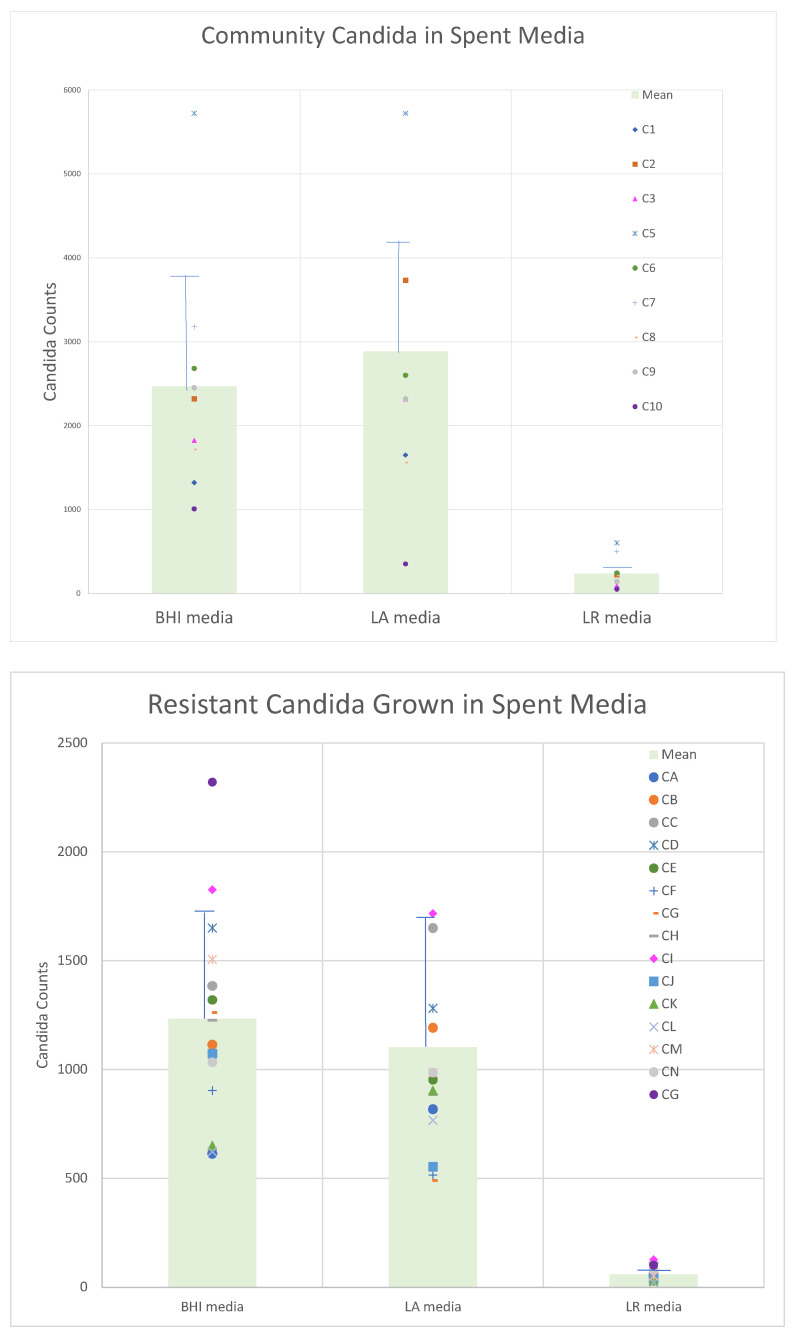
Spent (7-day-old) cultures of *Lactobacillus acidophilus* (LA) or *Lactobacillus rhamnosus* (LR) were inoculated with community isolate strains (upper panel) or strains from drug-resistant isolates panel of *Candida albicans* plus *Candida glabrata* (lower panel). *Candida* strains were grown overnight in brain heart infusion (BHI) broth and diluted 1:1000 and inoculated into 1 mL aliquots of filter-sterilized spent culture medium with an equal volume of BHI or fresh BHI. After overnight incubation, each culture was counted directly by flow cytometry (forward scatter events and reported as events/μL). Strains used in this experiment are abbreviated are described in more detail the methods section.

Having established the utility of flow cytometry for enumerating and differentiating the test organisms, we prepared cultures of each of the probiotic lactobacilli and incubated them for a total of 7 days, prior to recovering the spent medium. Filter-sterilized spent culture supernatants were inoculated with the panels of *Candida* to determine the relative ability of these supernatant fluids to support or inhibit yeast growth in comparison to fresh BHI broth. Figure 2 demonstrates that the growth yield of the drug-resistant organisms intrinsically (in fresh medium) had diminished growth potential compared to the community obtained organisms. These data also showed that the inhibitory activity obtained in the spent culture medium was, on average, greater for *L. rhamnosus* than for *L. acidophilus*.

Propidium iodide (PI) was used to assess membrane integrity as a surrogate for viability, based on dye exclusion. Evaluation of *Candida* incubated in spent medium from putative probiotic *Lactobacillus* revealed only modest, though detectable, effects on membrane integrity as almost all *Candida* isolates showed only as small increase in PI staining. Detailed findings are summarized in Table 1 which showed variable but detectable reduction in some of the spent cultures and again, *L. rhamnosus* appeared have a more consistent advantage over *L. acidophilus*.

### 2.2. Co-Culture Studies

A limitation of measuring the growth potential of opportunistic organisms in spent culture medium from probiotic organisms, is the possibility that effects of simultaneous cultivation of two species could involve interaction requiring direct contact between organisms. Use of flow cytometry to discriminate between yeast and bacteria provided a means to evaluate results obtained by co-cultivation of bacteria and yeast.

**Figure 3:**
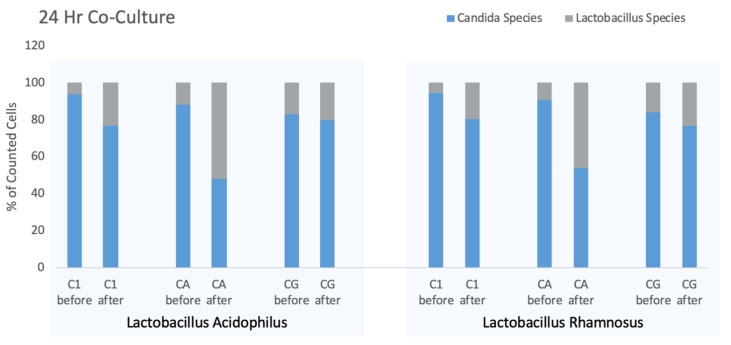
Six co-cultures were evaluated using 3 *Candida* strains paired with 2 *Lactobacillus* strains. Stacked bars represent 100% of the organisms in culture and the blue bars represent the proportion due to *Candida*. “Before” data represent the start of the experiment and “after” data are collected after 24 h at 37 °C. Each pair of bars represent a single culture comparing change during incubation.

For logistical reasons, a subset of the test organisms (additional details on strains is provided in the Methods) were used including CA-A, a multi-resistant strain from the MP8 collection (ATCC 64124), a community isolate CA-1 and the ATCC strain of *Candida glabrata* each paired with *L. acidophilus* or *L. rhamnosus*. Results were obtained from single cultures and replicates were not performed for this experiment with variation between strains being the point of emphasis. The results of this experiment showing the simultaneous counts of yeast and bacteria are presented in Figure 3. In this experiment, the ratio of yeast and bacteria were measured at the time of inoculation and then again after 24-h co-culture.

While the ratio of yeast to bacteria changed over the course of the co-culture, the counts of each strain of *Candida* and *Lactobacillus* showed individual changes. When incubated with *L. acidophilus,* the mean counts for three *Candida* strains declined from 708/μL to 497/μL whereas for *Candida* co-cultured with *L. rhamnosus* furnished an overall 2.2-fold increase in total counts (bacteria and yeast) from 578/μL to 1299/μL but with the *L. rhamnosus* increasing 4.6-fold during the incubation period.

In all cultures, the starting yeast count exceeded the number of bacteria, although considering the biomass of yeast compared to bacteria, the biomasses may have been more similar than particle counts would suggest. As noted above, the amplitude of the FSC parameter relates to the particle size and suggests that the biomass of ten bacteria approximates that of one yeast cell. After overnight incubation of each of the six cultures the proportion of the culture attributed to bacteria increased. Using paired T test to compare before and after percentages of all culture combinations the overall differences were significant (*p* = 0.0254), however, each combination appeared to have different levels of efficacy for *Lactobacillus* competition. The drug-resistant CA-A seemed most affected by the *Lactobacillus* strains, while *Candida glabrata* showed the smallest increment in *Lactobacillus* growth although these differences may not be significant.

These data suggest more extensive studies with more microbial strains would be warranted to gain a comprehensive picture of interspecies interactions may occur. However, the change in bacteria to yeast ratio over time seemed applicable to evaluate media supplements (such as prebiotics or inhibitory compounds) to alter the effect of lactobacilli on *Candida.*

**Figure 4:**
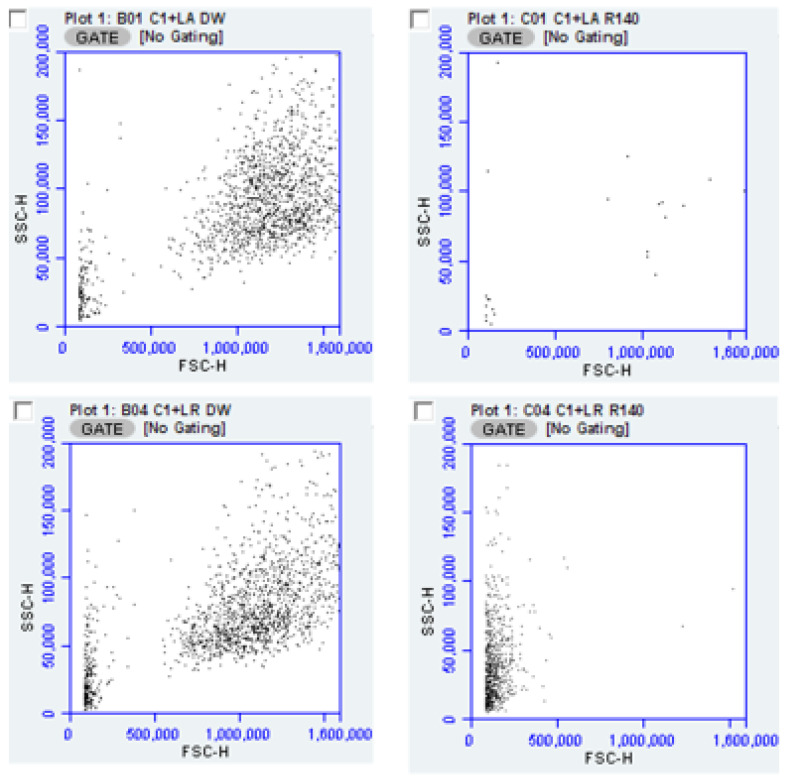
The effect of antimicrobial product, “R” added to co-cultures of yeast (CA-A, an antifungal drug resistant *Candida albicans* described further in the methods section.) and bacteria are indicated on the flow scatter plot as LA for *L. acidophilus* and LR for *L. rhamnosus*. Equal volumes of overnight cultures of *Lactobacillus* and *Candida* diluted 1:1000 prior to combining. Left panels show yeast-bacteria co-culture without “R” and the right panels show co-culture with “R” added. Bacteria in upper panels are *L. acidophilus* and lower panels, *L. rhamnosus*. All samples were diluted 1:10 before application to the flow cytometer.

The utility of flow cytometry for determining antimicrobial effects of products such as “R” is illustrated by the scatter plots below (Figure 4) depicting the decrease in overall counts after exposure to “R” for both *L. acidophilus* and *L. rhamnosus*, each cultured with CA-1. These data suggest a difference in the survivability of *L. rhamnosus* compared to *L. acidophilus* under these conditions.

To expand the translational value of these observations, we also applied the above experimental procedure to compounds that were part of the commercial preparation (“R”) or intended for use with it. Evaluation of the putatively active compounds is summarized in Table 2 which details the reduction in *Candida* counts in co-cultures with relevant additives. Additives noted below were mixed with equal volumes of BHI and inoculated with bacteria and yeast as before. “R” in separate studies (data not shown) proved highly inhibitory toward bacteria and yeast, and accordingly was prepared at an initial dilution of 1:40 in water (relative to the finished product supplied by the manufacturer). Other additives at 2% w:v included polydextrose, glycogen, crystalline bovine lactoferrin; disodium EDTA was 0.2% starting concentration and lactic acid was 4% starting concentration.

Data represents the *Candida* counts in co-cultures with stated additive/*Candida* counts in cognate co-culture without additive × 100 to indicate percent reduction compared to control. Candida strains employed in this experiment are described in greater detail in the methods section but includes CA-1, a clinical isolate, CA-A and CA-D multi-resistant isolates from the MP-8 panel from ATCC as well as an ATCC strain of *Candida glabrata*. Lactobacilli in this experiment were provided by Giellepi, SpA (see methods). Results for the four *Candida* strains were combined for each combination and aggregate data are presented.

The results of the addition of potential inhibitory compounds (“R”, EDTA, lactoferrin and lactic acid were anticipated to reduce the number of *Candida* organisms in co-culture above and decrease imposed by the presence of probiotic Lactobacilli. The complete “R” formulation at high dilution remained inhibitory and most of the other compounds did not, on average, prove as inhibitory as “R” which contains EDTA and lactic acid. The measures of data dispersion do indicate that for some inhibitors, substantial strain variation is evident. While not presenting the strains individually in Table 2, we observed that *Candida glabrata* produced higher numbers in co-cultures than the *C. albicans* strains and tended to be least susceptible to inhibitors.

Notable among observations from this experiment was the difference between the effect of polydextrose and glycogen. Both compounds are included in the formulation of “R” and are intended to serve as prebiotics to enhance the probiotic activity of *Lactobacillus*. While some effect was seen with polydextrose, the prebiotic effect associated with glycogen was profound and may be reflective of the commonly described role of glycogen in support of a benign vaginal microbiota.

Inhibitors added to co-cultures suppress all species in a mixed culture, but the data from these co-cultures with inhibitors was evaluated for the distribution of organisms in the yeast and bacterial fractions. The lactobacilli tended, as shown by the data in Table 3, to have good survivability in the face of inhibitory compounds and when the results for polydextrose and glycogen are compared, the findings are supportive of the likelihood that glycogen provides a stronger prebiotic effect than polydextrose.

### 2.3. Differential Viability of Co-Cultures

**Figure 5:**
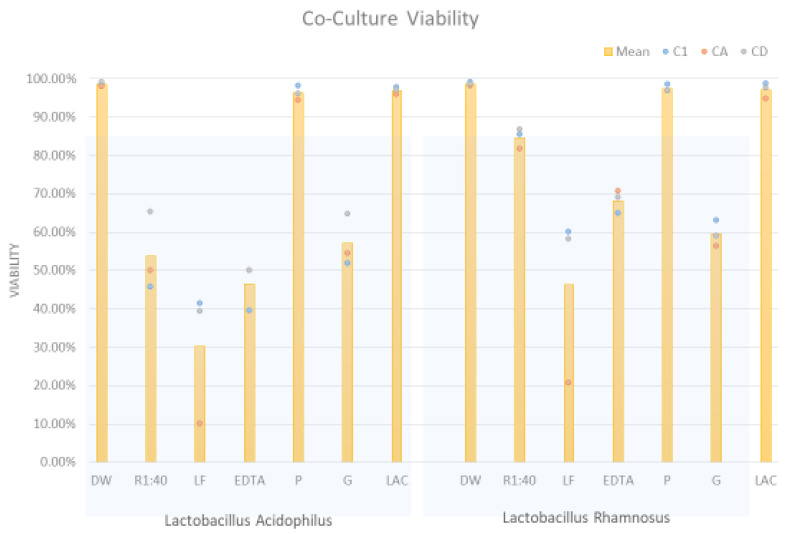
Overall propidium iodide exclusion of organisms in co-cultures of *Candida* and *Lactobacillus* showing the effect of antimicrobial and prebiotic additives. DW represents distilled water as a control for the volume of the other additives. These were at concentrations described in Table 2.

A further element of the flow cytometry data allowed estimation of the viability of organisms in co-culture experiments. Propidium iodide staining provides a measure of membrane compromise and PI exclusion serves as a surrogate for viability. Figure 5 shows the overall staining as a percent of the organisms in co-culture but does not discriminate between viability of *Candida* versus *Lactobacillus*. It was interesting that lactic acid did not appear to diminish the overall viability of the mixed cultures and likewise for polydextrose.

**Figure 6:**
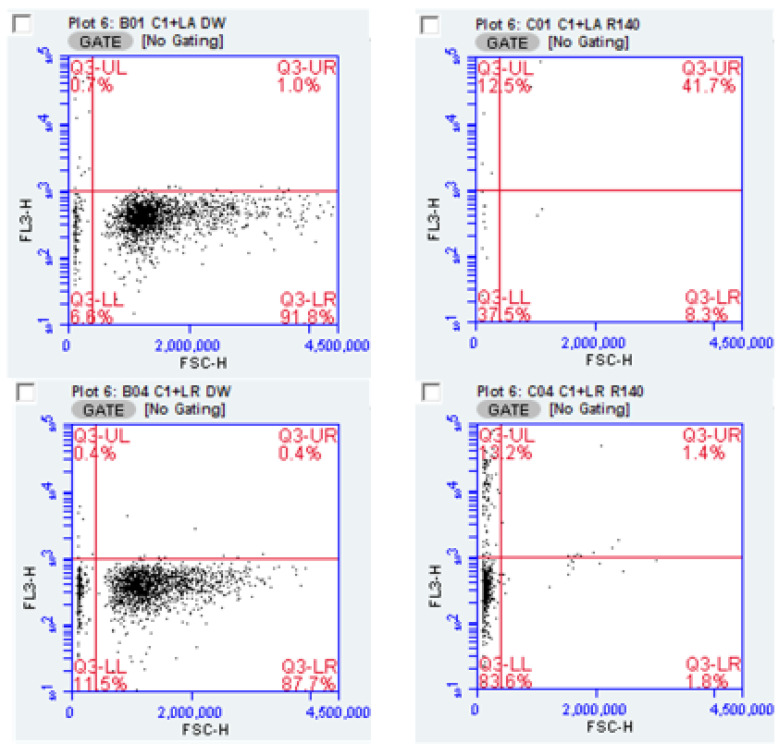
Flow cytometry of yeast-bacteria co-culture, with and without inhibitory additive “R”. Upper panels pair CA-1 with *L. acidophilus* and lower panels pair CA-1 with *L. rhamnosus*. Quadrant markers delineate propidium stained versus unstained on the FL3-h axis, while bacteria are distinguished from yeast on the FSC-h axis. Samples were diluted 1:10 prior to analysis, and due to scaling, some counted events are off the scale in this figure but recorded by the cytometer.

It may be argued that the number of counts attributable to *Candida* measured in co-culture are irrelevant if those counts were from non-viable organisms. Again, the evaluation of flow cytometry data was able to address this matter. Illustrating the approach is Figure 6 in which “R” is used as an exemplary inhibitor. With the forward scatter channel differentiating yeast from bacteria the orange fluorescence channel provided information on cell integrity.

Using this as an analytic approach, the percent viability of the *Candida* population exposed to lactobacilli and additives is presented in Table 4 addresses this issue. The data indicate the enhancement of inhibition of *Candida* was primarily associated with inhibitors from formulation “R”.

## 3. Discussion

The central hypothesis of this work was that flow cytometric evaluation of cultures of yeast and bacteria in co-culture could discriminate between counts due to yeast and bacteria and to simultaneously assess the membrane integrity of these cells. The potential of flow cytometry as a complementary tool for microbiology has been cited previously [7,8,9,10] and we have applied cytometric studies as enabling studies of multiple microbial species.

Three analytic parameters (forward scatter event counting, forward scatter impulse magnitude and fluorescence channel 3 were critical to the present work. These are, in order are importance to findings reported here, namely enumeration of particles (microorganisms) in the forward scatter channel. The cytometer used is quantitative, as the sample is introduced to the flow cell with a precision peristaltic pump as contrasted to pneumatic sample introduction in some other cytometers. The forward scatter channel values (peak height or area) relate to particle size which we have shown allows discrimination between yeast and bacteria. Finally, propidium staining detected in fluorescence channel 3, established the minimum number and proportion of non-viable organisms in each of the two co-cultured populations.

As an exemplar for addressing the central hypothesis a preliminary exploration of in vitro behavior of probiotic lactobacilli and their interaction with *Candida albicans* was undertaken in the context of a larger evaluation of the antimicrobial activity of a proprietary vaginal product (Respecta^®^ BalanceGel) and its potential for synergy with probiotic microorganisms. This topical vaginal anti-infective is reflective of the interest in developing non-antibiotic therapeutics based on natural components of the vaginal microenvironment, and which may be augmented by probiotic bacteria or prebiotics [11,12,13,14]. Since the goal of this study was to demonstrate the utility of the technology, more extensive experiments with larger panels of test organisms and antimicrobial substances could have been entertained if product development or head-to-head comparisons of probiotics were intended. However, with the limited purpose in view, we showed the adequacy of flow cytometry to simultaneously measure yeast and bacterial viability and numbers.

The translational value of this work is relevant to comparison of the culture-shaping activity of prebiotics and probiotics as well as the effect of inhibitory additives to defined cultures. In the present work, we were limited to study of yeast and bacteria in co-culture, which exploited the size difference between these species, but studies could be expanded to different bacterial species of similar physical dimensions with fluorescent tagged antibodies against specific microorganisms. Quantitative flow cytometric evaluation of the vaginal ecologic niche or other body sites could be readily developed, though we did not extend our work to that possibility at this time.

Probiotic studies that focus on inhibitory activity of spent culture fluids are relatively easy to perform and have been used to address probiotics related to gastrointestinal [15,16] or respiratory organisms [17]. However, monoculture in spent medium lacks the ability to mimic the interactions of multiple species cultured together in the same milieu. The ability to distinguish yeast from bacteria without resort to dilution studies on selective or differential media is advantageous and with the automation and relatively high throughput of flow cytometry the savings in human resources is notable. It is also important to note that previous reports indicated that bacteria (*E. coli*) could be counted by flow cytometry with a correlation coefficient of 0.99 compared to viable plate count [18]. In addition, the results from flow cytometry generate statistically relevant numbers of microorganisms counted and have the advantage of the availability of fluorescent stains which we have highlighted through use of the non-permeant vital stain, propidium iodide.

Some limitations of this work must be acknowledged, though their impact may be minor and could readily be addressed by follow-on detailed studies. With flow cytometry, the large number of individual events recorded for individual samples is large and generates statistical power that has allowed us to emphasize evaluation of multiple microbial strains rather than replicating individual cultures. In individual cultures we recorded 2000 counts per culture. As cultures were prepared in standard growth media, essentially all recorded counts necessarily were due to microorganisms inoculated into the media and unless the conditions of culture result in physical destruction of microorganisms, the physical organisms present in the starting culture will remain (viable or not) in the final sample to be evaluated. The microorganisms present in the inoculum would also contribute to the total count after growth occurs, the resulting counts will vastly overwhelm those present at inoculation. If, the culture conditions suppress growth, the final counts may largely be due to those present at inoculation. These considerations do not diminish the value of the method but demand care in evaluating the results. Plate counting techniques could provide similar data, especially if used with selective or differential media for co-culture experiments but are more labor intensive and less precise than cytometric measures and as noted above plate counts correlate well for a single species [18]. The only caveat for flow cytometry of microorganisms is that at high particle counts, coincidence (due to two particles simultaneously passing the FSC laser or due to aggregation of microorganisms in the sample) could lower counts. Accordingly, all samples were diluted ten-fold before analysis (see Methods) and vigorously vortexed just before introducing to the cytometer flow cell.

The present methods are not the only techniques useful in multi-species culture. In addition to standard plate-count techniques, molecular methods such as qPCR [19,20] could be used to quantify the relative abundance of yeast and bacteria together in a culture, but they are not appropriate for demonstrating the viability of the strains present in the experimental conditions, except that expansion of the population following overnight incubation, implies growth.

While the flow cytometric parameter reflecting particle size would not be expected to distinguish between two bacterial species, the availability of fluorescent Gram staining reagents may allow differentiation [21] however, two bacterial species having the same Gram reaction would require a taxon-specific fluor such as a labeled antibody.

The size difference between bacteria and yeast is readily revealed by forward scatter values in flow cytometry as has been documented here, and the availability of non-permeant fluorescent DNA stains allow for determining cellular integrity of both yeast and bacteria. This has proved tractable for determining the ratio of yeast to bacteria in co-culture and simultaneously, the membrane integrity of each subpopulation. While vital dye staining relies on loss of membrane integrity, other microorganisms could become non-viable for other reasons besides membrane damage. Thus, it is important not to assume that PI-stained cells must necessarily represent every non-viable organism in the sample.

An additional limitation of this study is that it is mainly an elaboration of a technique and some of the observations deserve deeper investigation. Altering the ratios of yeast to bacteria for the inoculation of cultures could generate varied results, and large doses of probiotic organisms could diminish their effectiveness if their numbers reach stationary growth phase levels early in the incubation period. Quorum sensing could limit the probiotic ability of *Lactobacillus* [22] in the case of a large inoculum, while a very small inoculum may fail to support probiosis. This theoretical possibility should be considered with intake of large oral inoculum of probiotic organisms and should be explored experimentally.

While the methods presented here have been valuable for preliminary studies, additional refinements may be considered. For example, there is some concern that injecting complex chemical and pharmaceutical compositions onto the cytometer might foul critical components of the instrument, but such concerns could be obviated by washing the cultures in buffer before applying them to the instrument. Recoveries after washing should be determined experimentally.

Although we had a larger number of test organisms available, for logistical reasons we did not evaluate every possible combination in this preliminary demonstration of responses among several combinations of yeast and bacteria. The ability of various inhibitors of interest to influence population dynamics in culture provided data that indicate some probiotic organisms may outperform others, especially when driven by a particular prebiotic. More combinations could have been explored; however, the analytic approach and how they may be applied in other settings was demonstrated, even with the limited number of combinations.

Interest continues in detailed mechanisms of how two or more populations interact over time, and some of these questions may be better approached through more sophisticated means such as bioreactor studies and transcriptomic studies, however, flow cytometric monitoring of timed samples from bioreactor studies would be useful. As a means of teasing out the contributions of various antimicrobial compounds to the overall antimicrobial activity of a formulated therapeutic product, the methods described here performed well and may be a prelude to further modifications of therapeutics.

Particularly interesting, is the use of prebiotics as part of a therapeutic or preventive composition and using the techniques presented here could allow a rational approach to selecting a particular probiotic organism as a lead for clinical use as well as pairing the probiotic organism with a prebiotic candidate.

Certainly, the most relevant information will be in clinical evaluation of antimicrobials, probiotics or prebiotics in a clinical setting. While some literature is available to support the concept of probiotics in such vaginal conditions as candidiasis [23] and bacterial vaginosis [24] the studies are still limited in both number and quality. Future clinical studies may be enhanced by rational selection of the parameters defined in vitro as we have presented here. The flow cytometric methods described do not have to be performed in isolation and the co-cultures are amenable to measurement of metabolites, virulence factors and biofilm among others.

## 4. Materials and Methods

*Candida albicans* isolates were obtained from two sources. The MP8 drug resistant *Candida albicans* panel was purchased from ATCC and consisted of 12 well-characterized strains with drug resistance to one or more azoles, echinocandins or flucytosine. Two additional strains in the MP8 panel are not drug resistant but are well-characterized isolates included for use as control cultures. In addition, our laboratory maintains 12 non-drug-resistant human (anonymous) isolates obtained from local clinical laboratories. In addition to the aforementioned organisms, an ATCC strain (2001) of *Candida glabrata* was also available. This organism was included because it is a common cause of non-albicans vaginitis and is considered particularly difficult to treat. Test organisms were selected from among the above strains in our culture collection.

Fungal organisms are grown in Sabouraud’s Dextrose Broth pH 6.8, aliquoted and frozen and stored at −80 °C. Prior to use they are thawed and grown as starter cultures overnight at 37 °C in Brain-Heart Infusion broth, pH 7.4, before use in experiments.

*Lactobacillus acidophilus* (strain GLA 14) and *Lactobacillus rhamnosus* (strain HN001) were provided by Giellepi SpA, Milan Italy, for use in testing prebiotic and probiotic activities of a proprietary topical gel formulation (Respecta^®^ BalanceGel, also abbreviated in this paper as “R”) also supplied by Giellepi.

Co-culture of *Candida* and *Lactobacillus* involves mixing equal volumes of starter cultures into a basal medium consisting of Brain Heart Infusion broth with an equal volume of water (control) or test article. The test articles employed in this study include the proprietary gel “R” and components of the gel with potential for influencing interaction between *Candida* and *Lactobacillus* which included: lactic acid, glycogen, polydextrose and disodium EDTA. Crystalline bovine lactoferrin was used in previous studies in conjunction with the probiotic lactobacilli [5,6].

Characterization of cultures employs flow cytometry using the BD Accuri C6 cytometer which has non-pneumatic sample injection mechanism allowing for absolute counts of particles, in this case, microorganisms. The cytometer is triggered in the forward scatter channel (FSC) to record each count. In addition, the peak height of the FSC signal is proportional to the particle size which allows discrimination between yeast and bacterial entities. While not used in this work, the FSC may be multiplied by the counts to provide an estimate of the biomass for a cell population.

For flow cytometric evaluation of cultures, growth media with and without additives is inoculated with combinations of yeast and bacteria and the counts of each are determined prior to overnight incubation based on forward scatter events and relative size of the organisms is determined by forward scatter height as illustrated in the results section.

At the conclusion of overnight incubation at 37 °C in 5% CO_2_, each culture is mixed well and a 100 uL aliquot is added to solution of propidium iodide in water (100 uL of a stock of propidium iodide at 60 mg/mL added to 50 mL of deionized water). Samples are evaluated by flow cytometry as noted above, and propidium iodide staining is also documented in the Fl3-h channel as a further parameter to indicate microbial cell integrity.

Raw data derived from multiple strains of yeast or bacteria were reduced by use of descriptive statistics (mean, range and standard deviation). Where multiple strains were used in the same experiment, biological effects such as growth, growth inhibition or viability data employed normalizing the results for individual strains which required dividing raw data by corresponding data from the appropriate control to establish percent change. The text identifies where this process was employed. In this report we did not make any comparisons of discrete parameters, but all of the comparisons were deemed to involve continuous data parameters. Discrete cells or particles are counted by the flow cytometer but counts and forward scatter height values are measured across a large (seven decades) dynamic range and behave more as a continuous rather than discrete variable, considered as justification of use of the T test. Likewise, the percent of yeast populations staining with propidium iodide as well as percent excluding propidium also represent continuous variables allowing use of T test as well. At each instance within the results section where T test was used, the text and figure or table legends state if paired or unpaired tests were employed. Comparisons yielding *p* < 0.05 were considered significant. Microsoft XL was used for evaluating data and calculating descriptive statistics and performing T tests.

## 5. Conclusions

Flow cytometry, because of its ability to report relative size of particles, is a quick and effective way to enumerate and differentiate yeast and bacteria in co-culture and was coupled with propidium iodide staining to provide information on integrity of the bacteria and yeast in culture. In addition to confirming the utility of flow cytometry, other key findings, such as that the probiotic effect of two *Lactobacillus* species was augmented by components of “R” and prebiotic compounds also augmented *Lactobacillus* probiotic activity and differences in probiotic activity between polydextrose and glycogen, were observed.

Experimental conditions may be altered to explore the intrinsic probiotic potential of lactobacilli or other potentially probiotic organisms. Culture conditions may also be manipulated to discover biological or chemical entities that push the dynamics of co-culture to favor probiosis over dysbiosis in artificial conditions which may have applicability to more complex in vivo scenarios. The differences in susceptibility of various pathogenic, opportunistic of dysbiosis-related microorganisms may be evaluated by these methods. The methods are also amenable to combinations with other kinds of analysis to provide broader insight to interspecies interactions.

## Figures and Tables

**Table 1:** Propidium iodide exclusion to demonstrate viability of yeast grown in spent culture supernatants from *Lactobacillus*.

	Percent PI Exclusion				
Test Organism	Growth Conditions	Mean % Unstained	Range	SD	Paired T Test (vs BHI) *p* =
Community Strains *n* = 13 *	BHI	94.6	90.4–96.8	0.12	-
	*L. acidophilus*	95.7	91.3–97.4	0.14	0.07
	*L. rhamnosus*	88.0	77.0–93.8	0.14	0.004
Drug-resistant Strains ** n = 12	BHI	88.2	47.8–95.9	0.02	-
	*L. acidophilus*	90.0	43.2–97.0	0.02	0.03
	*L. rhamnosus*	83.9	44.4–94.	0.05	0.07

* Included with Community Strains were 2 non-drug resistant controls from the MP8 panel. ** *C. glabrata* was included with the drug resistant panel of organisms.

**Table 2:** Decrement in *Candida* exposed to co-culture additives.

	Percent Reduction with Compounds Added to Co-Cultures Compared to Control					
	Mean (SD) Range					
	“R”	Polydextrose	Glycogen	EDTA	Lactoferrin	Lactic Acid
*Candida* strains co-cultured with *L. acidophilus*	73.3(5.8) 64.8–79.4	21.9 (11.9) 9.5–39.5	91.8 (4.6) 85.3–95.7	73.2 (4.9) 65.8–79.3	44 (21.3) 14.5–73.9	49.1(22.5) 22.7–77.8
*Candida* strains co-cultured with *L. rhamnosus*	80.5 (13.5) 58–93.9	19.4 (15.4) 6.4–44	70.5 (4.0) 64.9–74.2	59.7(25.5) 15.8–77.5	52.8 (6.0) 44–60.8	55.5 (5.9) 48.5–62.9

**Table 3:** Percent of co-cultures attributable to *Lactobacillus.*

	*Lactobacillus* as Percent of Co-Culture						
	Mean (SD)						
	*Lactobacillus* Starting Population	“R”	Polydextrose	Glycogen	EDTA	Lactoferrin	Lactic Acid
*L. acidophilus*	7.1 (0.3)	69 (13.8)	16.9 (6.3)	92.5 (22.2)	74.7 (5.6)	39.9 (32.0)	52.7 (20.9)
*L. rhamnosus*	18.6 (8.6)	94.8 (0.2)	42.2 (13.5)	75.8 (9.1)	85.1 (3.5)	51.9 (0.3)	63.4 (8.0)

**Table 4:** Viability of *Candida* in Co-culture with lactobacilli (% excluding propidium iodide).

Additive	*Lactobacillus acidophilus*			*Lactobacillus rhamnosus*		
	CA-1	CA-A	CA-D	CA-1	CA-A	CA-D
DW (BHI control)	98.92	99.57	99.79	99.55	99.12	98.79
“R” 1:40	16.60	40.09	80.21	56.25	56.10	58.54
Lactoferrin	22.37	1.05	33.88	63.92	5.88	58.06
EDTA	11.17	50.00	62.56	12.67	14.17	14.85
Polydextrose	98.66	98.12	97.74	99.23	97.71	96.20
Glycogen	59.20	40.54	40.54	79.47	68.45	71.30
Lactic Acid	98.60	89.55	97.57	98.98	88.58	96.42
